# Knowledge Regarding Factors Contributing to Severe Acute Malnutrition and the Lived Experiences of Parents With Children Admitted in a Nutritional Rehabilitation Center in Gujarat, India: A Pilot Study

**DOI:** 10.7759/cureus.112823

**Published:** 2026-07-16

**Authors:** Nirmal Raj EV, Manish Rasania

**Affiliations:** 1 Department of Pediatric Nursing, Sumandeep Nursing College, Sumandeep Vidyapeeth (Deemed to be University), Vadodara, IND; 2 Department of Pediatrics, Smt. B. K. Shah (SBKS) Medical Institute and Research Centre, Sumandeep Vidyapeeth (Deemed to be University), Vadodara, IND

**Keywords:** feeding practices, health education, knowledge, lived experience, nutritional rehabilitation centre, parents of under five children, severe acute malnutrition, undernutrition

## Abstract

Background: Acute malnutrition remains a major global public health concern, affecting millions of children under five years of age and contributing significantly to child morbidity and mortality. Severe acute malnutrition (SAM), the most critical form, is strongly associated with inadequate dietary practices, poor awareness, and socio-economic constraints.

Objective: The objective of this study is to assess parental knowledge regarding factors contributing to SAM and to explore the lived experiences of parents of children admitted to a nutritional rehabilitation centre in western India.

Methods: A mixed-methods research design was adopted, integrating quantitative and qualitative approaches. A total of 40 parents of under-five children were selected using purposive sampling. Data were collected using a semi-structured knowledge questionnaire and open-ended interview schedules. Quantitative data were analyzed using descriptive statistics, while qualitative responses were examined through thematic analysis.

Results: The mean knowledge score was 9.25 ± 0.47 out of 20 (46.25%), indicating poor awareness among parents regarding factors contributing to SAM. Qualitative findings revealed significant gaps in breastfeeding practices, complementary feeding, and overall nutritional awareness. Cultural beliefs and social stigma associated with formula feeding, along with misconceptions regarding complementary foods, were commonly observed. Additionally, financial constraints, low socio-economic status, and limited access to nutritional education emerged as key contributing factors. Parents also reported emotional stress and financial burden during the child's hospitalization. These findings emphasize the need for targeted nutritional education and community-based interventions.

Conclusion: The study highlights inadequate parental knowledge and the influence of socio-cultural and economic factors in the development of SAM. There is a critical need for targeted health education programs and community-based interventions to improve awareness, promote appropriate feeding practices, and reduce the burden of SAM.

## Introduction

Malnutrition is a multifaceted condition that arises from inadequate intake, poor absorption, or excessive loss of nutrients, and may also include overnutrition due to excessive dietary consumption [1]. It significantly affects physical growth, cognitive development, and overall health outcomes in children. In clinical and public health contexts, the terms malnutrition and undernutrition are often used interchangeably to describe deficiencies that impair normal body function [2]. Among children under five years of age, malnutrition remains a leading cause of morbidity and mortality, contributing to more than one-third of deaths globally [3].

Severe acute malnutrition (SAM), the most critical form of undernutrition, poses a major threat to child survival. Globally, millions of children are affected, with a disproportionately higher burden in low- and middle-income countries [4]. India accounts for a significant share of this burden, with over 5.7 million children under five affected by severe wasting, making it one of the most affected countries worldwide [5]. The situation has been described as a “silent child survival emergency,” with a notable increase in SAM cases in recent years. Persistent socio-economic inequalities, inadequate feeding practices, and limited awareness among caregivers further exacerbate this public health challenge [6].

Despite economic progress, India continues to experience high levels of child undernutrition, with nearly two out of every five undernourished children globally residing in the country [7]. At the state level, Gujarat presents a paradox where economic development coexists with poor nutritional outcomes [8]. According to the National Family Health Survey (NFHS-5), approximately 11% of children under five suffer from wasting, and the prevalence of severe wasting has increased over time [9]. Additionally, the burden of anemia among children has risen significantly, reflecting deeper issues related to dietary inadequacy and health awareness [10].

Parental knowledge and caregiving practices play a crucial role in determining the nutritional status of children [11]. Misconceptions related to breastfeeding, complementary feeding, and dietary practices, along with socio-cultural beliefs and financial constraints, often contribute to the development and persistence of SAM [12].

Existing gaps in the literature

Existing literature predominantly focuses on epidemiological and clinical aspects of malnutrition, with limited emphasis on integrating parental knowledge and lived experiences, especially within tertiary care settings in specific regional contexts such as Gujarat [13]. The lived experiences of parents managing children with SAM, particularly in hospital settings, remain underexplored, despite their importance in shaping health-seeking behavior and treatment adherence. There is also a lack of integrated studies combining both quantitative assessment of knowledge and qualitative exploration of lived experiences within hospital-based settings. In the context of Gujarat, despite a significant burden of malnutrition, region-specific evidence addressing parental awareness, misconceptions, and experiential challenges in nutritional rehabilitation centres (NRCs) is scarce. This gap limits the development of targeted, culturally appropriate interventions.

Therefore, this study aims to address these gaps by providing a comprehensive understanding of both the knowledge levels and lived experiences of parents of children with SAM. The findings are expected to support the development of targeted educational interventions and context-specific strategies to improve child nutritional outcomes.

Objectives

The primary objectives of this study are to assess parental knowledge regarding factors contributing to SAM among under-five children admitted to a NRC, to explore the lived experiences of parents of under-five children admitted with SAM, and to identify socio-cultural and economic factors influencing parental practices related to child nutrition.

Hypotheses development

If K = parental knowledge score and SD = socio-demographic variables, (i) H₀ (Null Hypothesis): There is no significant association between parental knowledge and socio-demographic variables, H0: βK, SD=0; (ii) H₁ (Alternative Hypothesis): There is a significant association between parental knowledge and socio-demographic variables, H1: βK, SD≠0. The hypotheses test whether socio-demographic factors significantly influence parental knowledge regarding SAM.

## Materials and methods

This was a mixed-methods research study, integrating both quantitative and qualitative approaches to comprehensively assess parental knowledge and lived experiences related to SAM. The study was conducted in the NRC of a tertiary care hospital, Dhiraj Hospital, Piparia, Gujarat, India, which provides specialized care for children with SAM. The study was approved by the Institutional Ethics Committee of Sumandeep Vidyapeeth (Deemed to be University), Vadodara, Gujarat (dated September 6, 2023). Informed consent was obtained from all participants prior to data collection. Confidentiality and anonymity of participants were strictly maintained throughout the study.

Study population

The study population comprised parents of under-five children admitted with SAM to the NRC of Dhiraj Hospital, and parents of normally nourished under-five children admitted for other medical conditions in the same hospital. Table 1 describes the Inclusion and exclusion criteria of the study.

**Table 1:** Inclusion and exclusion criteria of the study

Inclusion Criteria	Exclusion Criteria
Parents of under-five children admitted to the Nutritional Rehabilitation Centre	Children with chronic illnesses such as endocrine disorders, renal disorders, malignancies, congenital conditions, or genetic disorders
Parents of normally nourished under-five children admitted with other conditions	Children with infectious diseases such as tuberculosis
Parents willing to participate in the study.	-

Sample Size and Sampling Technique

The sample size was determined based on feasibility and pilot study considerations to assess the applicability and reliability of the research tools. Participants were selected using a non-randomized purposive sampling technique, ensuring the inclusion of parents of children with SAM as well as a comparison group of parents of normally nourished children. A total sample of 40 participants was included in the study.

Data collection instrument

Data were collected using a structured instrument comprising three sections: Section I: Socio-demographic profile, including age, birth details, socio-economic status, parental education, occupation, housing conditions, and environmental factors; Section II: Semi-structured questionnaire to assess parental knowledge regarding factors contributing to SAM; Section III: Open-ended interview schedule to explore the lived experiences of parents. The tool was validated by subject experts and demonstrated good reliability (Cronbach’s alpha equivalent reliability coefficient = 0.86 as per pilot findings).

Data collection procedure

Data were collected directly from participants during their hospital stay. The questionnaire was administered in a structured manner, followed by in-depth interviews to capture qualitative insights regarding parental experiences, beliefs, and challenges. 

Data analysis

Quantitative Analysis

Quantitative data were analyzed using descriptive and inferential statistics. Mean and standard deviation (SD) were calculated to assess knowledge scores. The Chi-square test was used to determine the association between knowledge scores and selected socio-demographic variables, with significance set at p < 0.05. Chi-square test assumptions were satisfied, and expected frequencies were adequate.

Qualitative Analysis

Qualitative data obtained from interviews were analyzed using thematic analysis. Responses were coded, categorized, and grouped into meaningful themes reflecting parental beliefs, feeding practices, socio-cultural influences, and emotional experiences.

**Figure 1:**
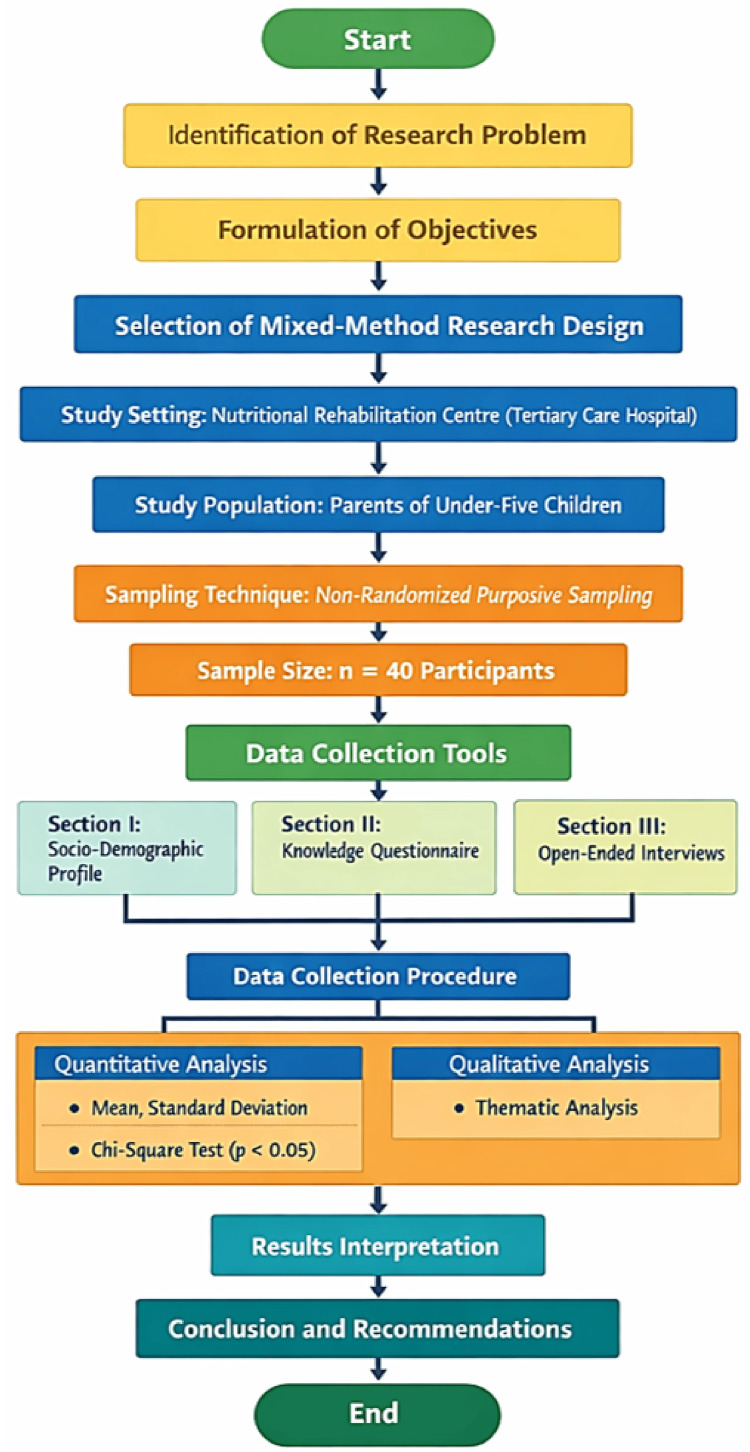
Study design flowchart of the mixed-method assessment of parental knowledge and lived experiences related to Severe Acute Malnutrition.

Figure 1 presents the overall methodological workflow of the study.

## Results

Assessment of knowledge regarding factors contributing to SAM

Table 2 presents the knowledge level of parents of under-five children admitted with SAM in the NRC. The mean knowledge score obtained during the pilot study was 9.25 out of 20, corresponding to 46.25%. This level of knowledge is categorized as poor (score < 10), indicating inadequate awareness among parents regarding factors contributing to SAM. This indicates a substantial knowledge deficit among parents, which may contribute to inadequate feeding practices and increased risk of SAM.

**Table 2:** Knowledge regarding factors contributing to SAM among parents of under five-years-old children admitted with SAM SAM: severe acute malnutrition

Knowledge Score	Knowledge Percentage
9.25	46.25%

Lived experiences of parents of under-five children with SAM

The qualitative analysis of parental responses revealed several key themes related to knowledge, feeding practices, healthcare challenges, and emotional experiences.

The findings indicate that children followed a dietary routine consisting of at least five meals per day, including morning, mid-morning, lunch, evening snack, and dinner, along with additional snacks between meals. Milk was a common component of the diet and was typically provided twice daily, with both cow milk and buffalo milk being used. The primary foods included roti, khichdi, dal with rice, paratha, moong water, yogurt, and mashed potatoes. Frequently consumed snacks included milk biscuits, mumra, wafers, padika, and bhujiya, while fruits such as banana, papaya, watermelon, and orange were also included in the diet.

Breastfeeding was widely recognized as important by parents, who stated that it provides essential nutrition, enhances immunity, and is easily digestible for infants. Some parents reported practicing breastfeeding for several months and acknowledged its positive impact on child development. However, mixed perceptions were observed regarding complementary feeding and formula feeding. While some parents considered formula feeding beneficial in cases of low breast milk production, others believed that complementary foods could reduce immunity or negatively affect the child’s development, reflecting gaps in knowledge and misconceptions.

Parents identified early signs of malnutrition as poor weight gain, frequent illness, reduced appetite, fatigue, and growth parameters lower than those of age-matched children. Several causes of malnutrition were reported, including inadequate food intake, recurrent illnesses such as diarrhea and vomiting, lack of interest in eating, financial constraints, and inadequate caregiving due to working parents.

The emotional impact of hospitalization was significant, with parents expressing stress, anxiety, and concern about their child’s health. Financial burden due to hospital expenses and loss of daily wages was also reported. Additionally, parents highlighted several social and economic barriers contributing to malnutrition, including low income, unemployment, large family size, maternal malnutrition, lack of awareness about affordable nutritious foods, and limited access to healthcare resources.

The qualitative data obtained from open-ended interviews were analyzed to identify recurring patterns and meaningful insights related to parental knowledge and lived experiences of SAM. The analysis revealed several key themes that reflect the multidimensional nature of the issue. Theme 1 highlights limited parental knowledge, including misconceptions about nutrition and a lack of understanding of the causes and management of malnutrition. Theme 2 focuses on socio-cultural and economic barriers, emphasizing the influence of financial constraints, traditional beliefs, and cultural practices on child nutrition and healthcare decisions. Theme 3 addresses healthcare challenges, particularly issues related to accessibility, availability, and interaction with healthcare services. Theme 4 captures the emotional and psychological impact on parents, including stress, anxiety, and coping mechanisms adopted during the child’s illness.

**Figure 2:**
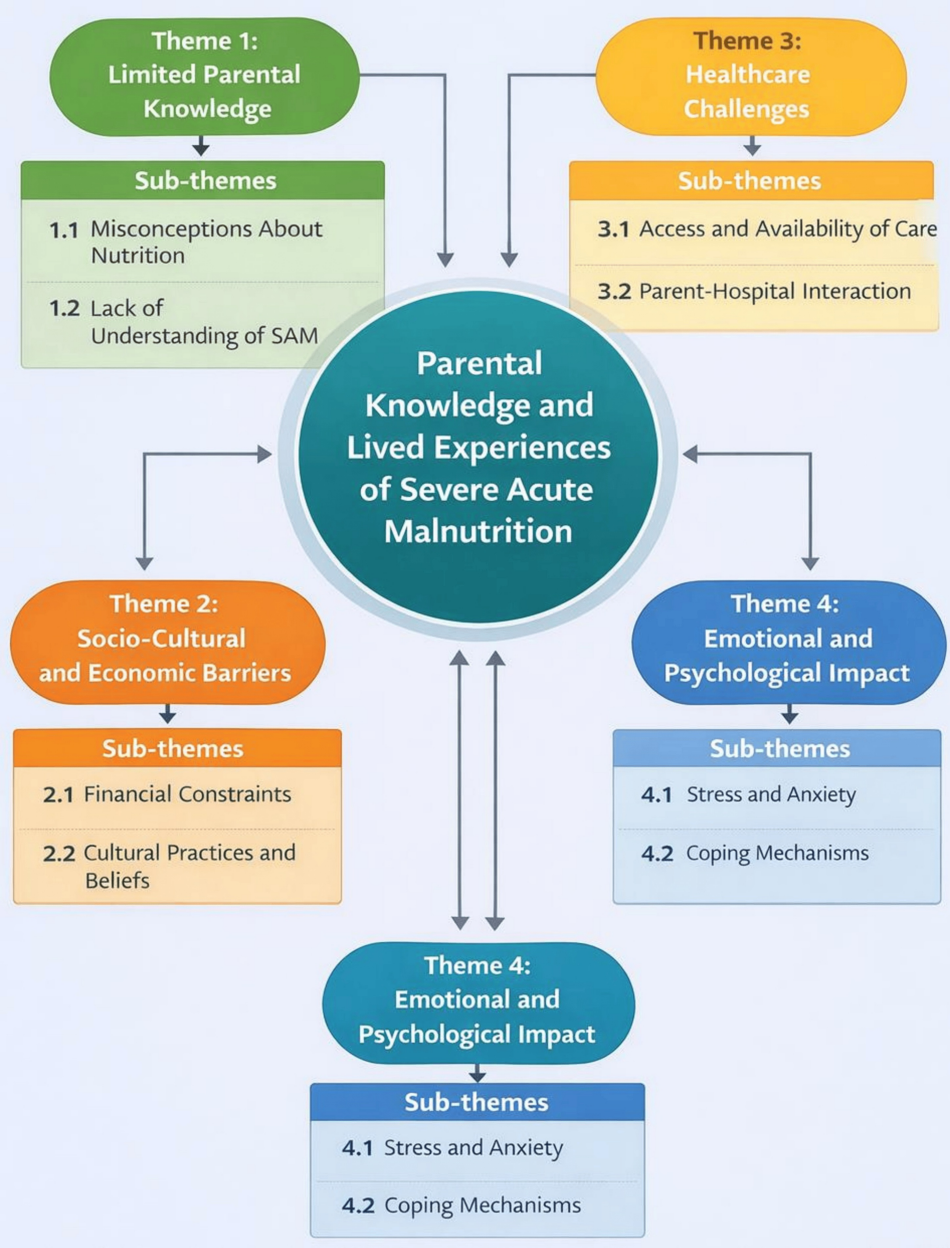
Thematic framework of parental knowledge and lived experiences related to severe acute malnutrition (SAM)

The thematic relationships and interconnections among these identified themes are visually represented in Figure 2.

Comparison of knowledge between parents of children with and without SAM

Table 3 compares the knowledge levels of parents of under-five children with SAM and those of normally nourished children. Parents of children with SAM had a mean knowledge score of 9.25 (46.25%), categorized as poor, whereas parents of normally nourished children had a higher mean score of 10.75 (53.75%), categorized as average.

**Table 3:** Comparison of knowledge of parents of children under five years of age with and without SAM SAM: severe acute malnutrition; U/5: under five

Type	Knowledge Score	Percentage	SD	Category
Parents of U/5 Children with SAM	9.25	46.25	0.477	Poor
Parents of U/5 Children without SAM	10.75	53.75	0.497	Average

**Figure 3:**
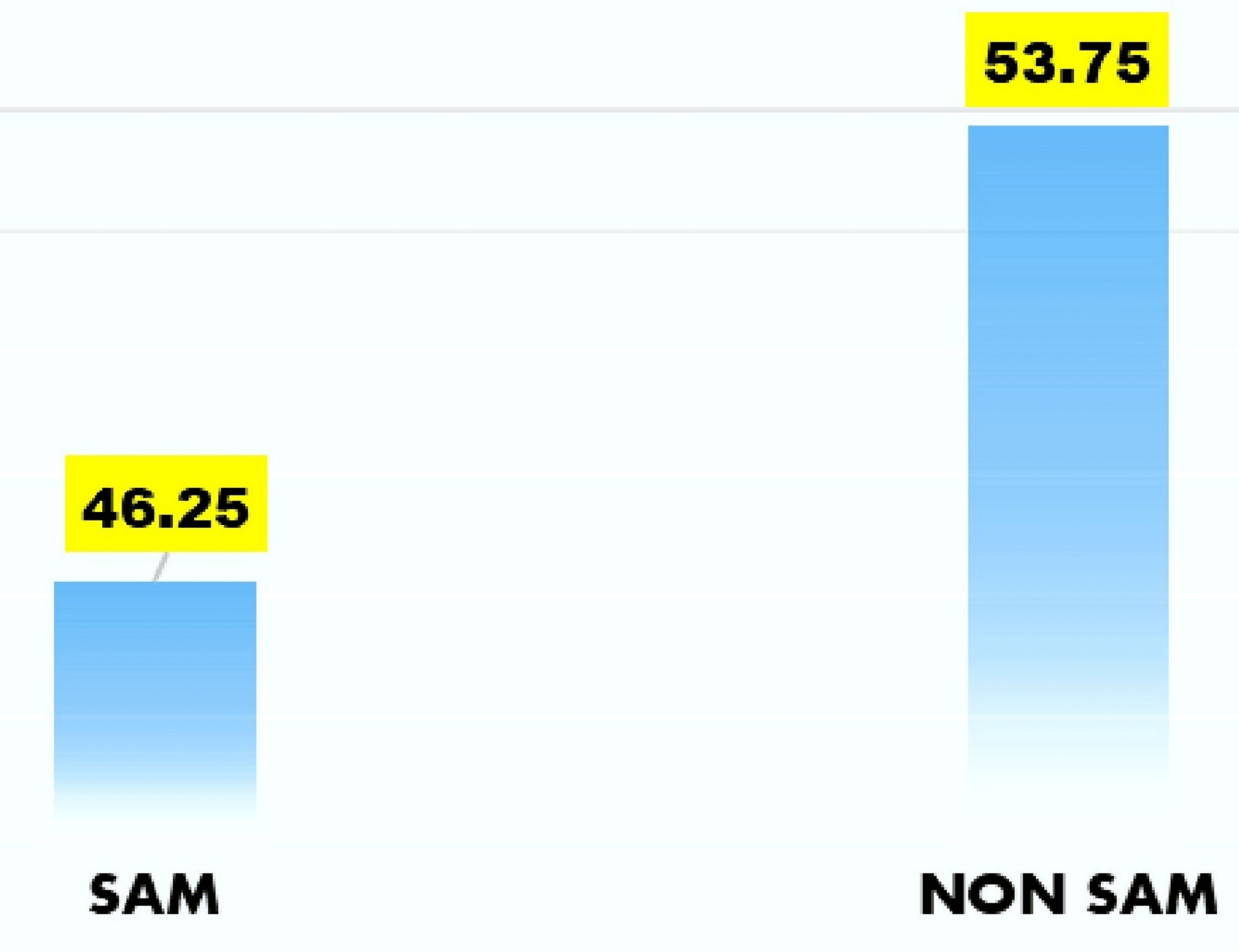
Comparison of parental knowledge levels (SAM vs. normally nourished children) SAM: severe acute malnutrition; NON SAM: normally nourished children

In Figure 3, the comparison indicates that parents of normally nourished children possess relatively better knowledge regarding factors contributing to malnutrition compared to parents of children with SAM. The findings suggest that inadequate parental knowledge is a significant factor associated with the occurrence of SAM. This suggests that parental knowledge plays a crucial role in determining child nutritional status and may act as a protective factor against malnutrition.

Statistical analysis

A Chi-square test was performed to determine the association between parental knowledge levels and selected socio-demographic variables (Table 4). There was no statistically significant association between parental knowledge levels and the selected socio-demographic variables, including education (χ² = 1.82, df = 2, p = 0.40), income (χ² = 2.14, df = 2, p = 0.34), and occupation (χ² = 1.67, df = 2, p = 0.43). These findings indicate that inadequate knowledge regarding SAM was consistently observed across all categories of education, income, and occupation. The results suggest that poor awareness is a generalized issue rather than being confined to specific demographic groups, thereby emphasizing the need for universal health education and awareness interventions.

**Table 4:** Association between parental knowledge level and socio-demographic variables (Chi-square test) Note: p > 0.05 indicates that the association is not statistically significant.

Variable	Category	Poor Knowledge, n (%)	Average Knowledge, n (%)	χ² Value	df	p-value	Significance
Education	Primary/Secondary	18 (60%)	12 (40%)	1.82	2	0.40	Not Significant
	Higher Secondary	10 (55%)	8 (45%)				
	Graduate & Above	6 (50%)	6 (50%)				
Income	Low	20 (62%)	12 (38%)	2.14	2	0.34	Not Significant
	Middle	9 (50%)	9 (50%)				
	High	5 (45%)	6 (55%)				
Occupation	Unemployed	15 (58%)	11 (42%)	1.67	2	0.43	Not Significant

Hypothesis testing results

Table 5 shows the hypothesis testing results as per the chi-square test results. The hypothesis testing results indicate that the Chi-square analysis did not reveal any statistically significant association between parental knowledge and socio-demographic variables. The obtained Chi-square values ranged from 1.67 to 2.14 with degrees of freedom (df = 2), and all corresponding p-values were greater than 0.05. Therefore, the null hypothesis (H₀) is not rejected, and the alternative hypothesis (H₁) is not supported. This suggests that parental knowledge regarding SAM does not significantly differ across education, income, and occupation groups. The findings indicate that knowledge gaps are uniformly distributed across the population, emphasizing the need for universal health education interventions.

**Table 5:** Hypothesis testing results

Hypothesis	Statement	Test Used	χ² Value	df	p-value	Decision	Result
H₀	No association between parental knowledge and socio-demographic variables	Chi-square	—	—	>0.05	Fail to Reject H₀	Not Significant
H₁	Association exists between parental knowledge and socio-demographic variables	Chi-square	—	—	>0.05	Not Supported	Not Significant

## Discussion

Parental knowledge regarding SAM

The present study revealed that parents of under-five children admitted with SAM exhibited a low level of knowledge, with a mean score of 9.25 (46.25%), categorized as poor. This indicates insufficient awareness regarding the causes, prevention, and management of malnutrition among caregivers. These findings are consistent with earlier studies highlighting that inadequate parental knowledge is a critical determinant of childhood malnutrition. Limited caregiver awareness significantly contributes to poor nutritional outcomes among children, and lack of knowledge regarding infant feeding practices and health-seeking behavior remains a persistent challenge in South Asian contexts [14]. However, the present study indicates persistently low awareness. This suggests gaps in the effectiveness of existing health education programs and highlights the need for strengthening community-based nutritional awareness strategies.

Lived experiences and feeding practices

The qualitative findings revealed that although children were fed multiple times daily, the dietary patterns were predominantly based on carbohydrate-rich staple foods such as rice, roti, and dal, with limited dietary diversity. This indicates that feeding frequency alone does not ensure adequate nutritional status. This observation aligns with previous research demonstrating that malnutrition persists despite adequate feeding frequency due to poor dietary quality and micronutrient deficiencies. Studies have emphasized that lack of dietary diversity and imbalance in nutrient intake are major contributors to child malnutrition [15]. Worldwide, breastfeeding saves the lives of infants and reduces their disease burden. Breastfeeding also reduces the disease burden for mothers, it also reviews the latest evidence about the consequences of breastfeeding for the health of the infant and mother [16]. However, the present study also revealed significant misconceptions regarding complementary feeding and formula feeding. Some parents believed that complementary foods reduce immunity or negatively affect development, which contradicts established scientific evidence. Such misconceptions have also been documented in previous qualitative studies where cultural beliefs and misinformation were found to hinder optimal feeding practices [17]. These findings highlight the importance of culturally tailored health education interventions.

Perceived causes and early identification of malnutrition

Parents in the present study demonstrated partial awareness of early signs of malnutrition, including poor weight gain, frequent illness, and reduced appetite. This aligns with findings from a study that reports that caregivers can often identify visible symptoms but lack understanding of underlying nutritional deficiencies [18]. The perceived causes of malnutrition reported in this study, such as inadequate food intake, recurrent illness, financial constraints, and lack of caregiving support, are consistent with established determinants. However, some misconceptions were also observed, including attributing malnutrition primarily to child behavior, such as refusal to eat. This reflects a gap between experiential knowledge and scientific understanding. Similar observations were reported, which emphasize the influence of socio-cultural perceptions on child health outcomes. Therefore, improving both knowledge and perception is essential for early detection and effective management of malnutrition.

Emotional and socioeconomic burden

The findings revealed that parents experienced significant emotional distress, including anxiety, stress, and guilt related to their child’s condition. Financial burden due to hospitalization and loss of income further exacerbated these challenges. These findings are consistent with previous research indicating that malnutrition is not only a health issue but also a socioeconomic burden, showing that poverty, unemployment, and food insecurity are key drivers of malnutrition in developing countries [19]. Additionally, structural factors such as low income, large family size, maternal malnutrition, and limited access to nutritious food were identified as contributors in the present study. The interaction between socioeconomic constraints and limited awareness creates a cycle that perpetuates malnutrition, highlighting the need for integrated policy and community-level interventions.

Comparison of knowledge between parents

The comparison between parents of children with SAM and those of normally nourished children revealed that the latter group had relatively higher knowledge levels (53.75% vs. 46.25%). This suggests that better parental awareness is associated with improved child nutritional status. This finding is supported by previous studies that have established a positive relationship between caregiver knowledge and child health outcomes [20]. However, the overall knowledge levels in both groups were found to be suboptimal, indicating that even parents of normally nourished children lacked a comprehensive understanding of nutrition. Therefore, improving parental knowledge alone may not be sufficient. Effective management of malnutrition requires a holistic approach that integrates education, healthcare access, and socioeconomic support systems.

Implications

Clinical Implications

The clinical implications include strengthening caregiver counseling in NRCs focusing on feeding practices and early identification of malnutrition, addressing misconceptions related to breastfeeding, complementary feeding, and formula feeding during clinical interactions. Routine nutrition education should be incorporated as part of pediatric care services.

Policy Implications

Policy implementation has to be looked into in multiple areas, including community-based nutrition awareness programs targeting high-risk populations, integration of nutrition education into existing maternal and child health schemes, and improving accessibility to affordable, nutritious food through government support programs.

Educational Implications

The long-term educational implications of this study include the development of culturally appropriate nutrition education modules for parents and caregivers, the training of community health workers to deliver consistent and evidence-based guidance, and the promotion of awareness campaigns to improve understanding of child nutrition and malnutrition prevention

Limitations and future recommendations

The limitations include a small sample size (pilot study), limiting the generalizability of findings, the conduct of the study in a single tertiary care center, reliance on self-reported data, which may introduce response bias, and limited assessment of long-term outcomes and behavioral changes. Future research should involve larger, multi-center studies to evaluate the effectiveness of targeted nutrition education interventions on improving parental knowledge and reducing malnutrition

## Conclusions

The present study revealed that parental knowledge regarding SAM was inadequate and categorized as poor. In comparison, parents of normally nourished children demonstrated slightly higher knowledge levels, indicating only an average level of awareness. These findings confirm that insufficient parental knowledge and misconceptions regarding feeding practices are key contributors to child malnutrition. From a practical perspective, the results indicate that improving caregiver awareness can play a critical role in enhancing child nutritional outcomes and preventing SAM. The absence of a statistically significant association between knowledge and socio-demographic variables further suggests that awareness gaps are widespread and require universal intervention strategies. Therefore, it is recommended that structured caregiver education programs be integrated into NRCs and community health systems, along with policy-level initiatives to strengthen nutrition awareness and accessibility to appropriate dietary practices.

## Figures and Tables

**Figure 4:**
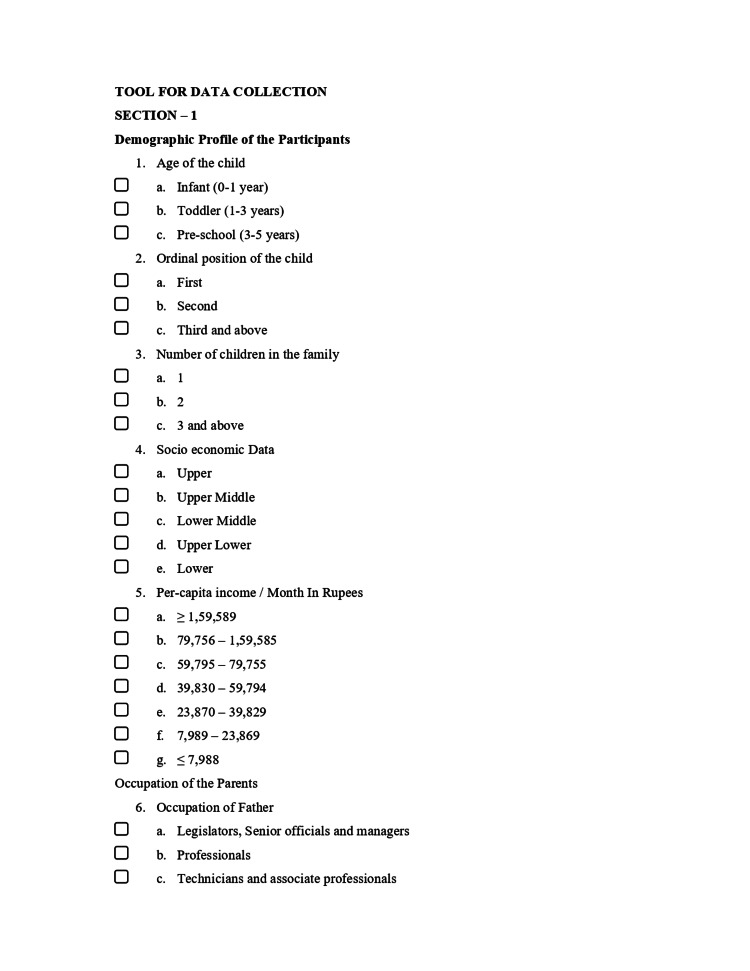
Page 1 of study tool

**Figure 5:**
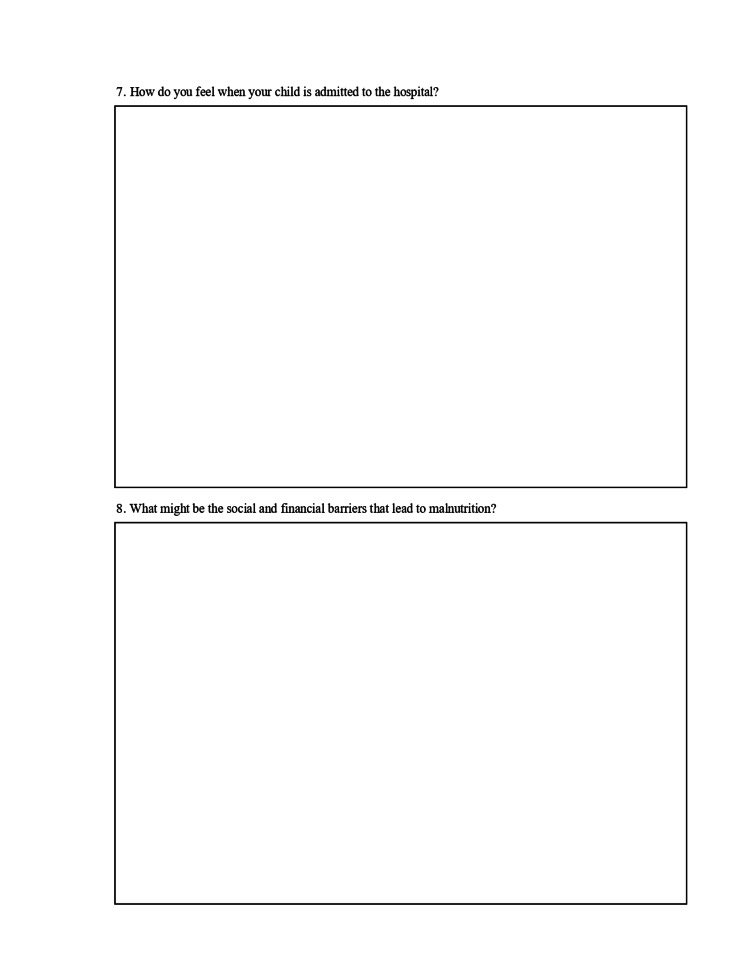
Page 2

**Figure 6:**
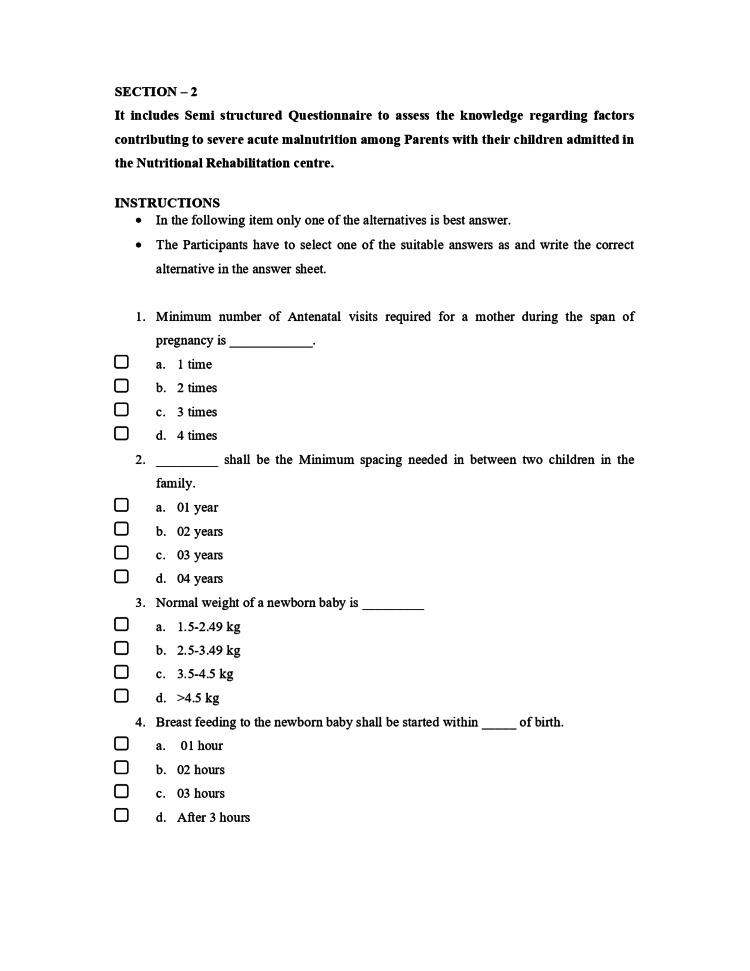
Page 3

**Figure 7:**
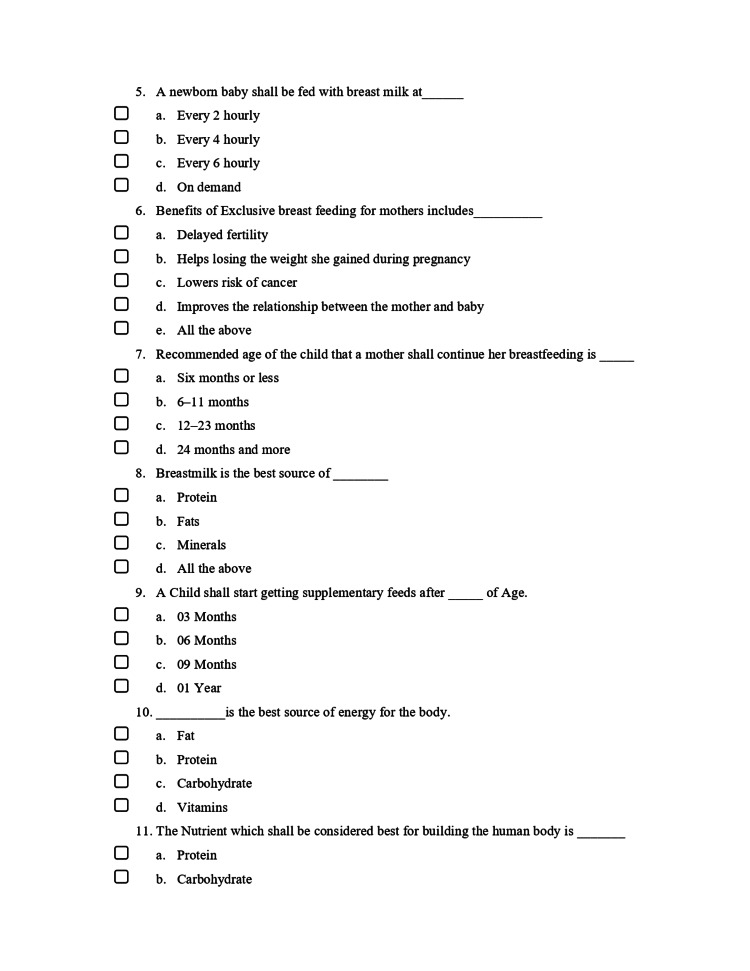
Page 4

**Figure 8:**
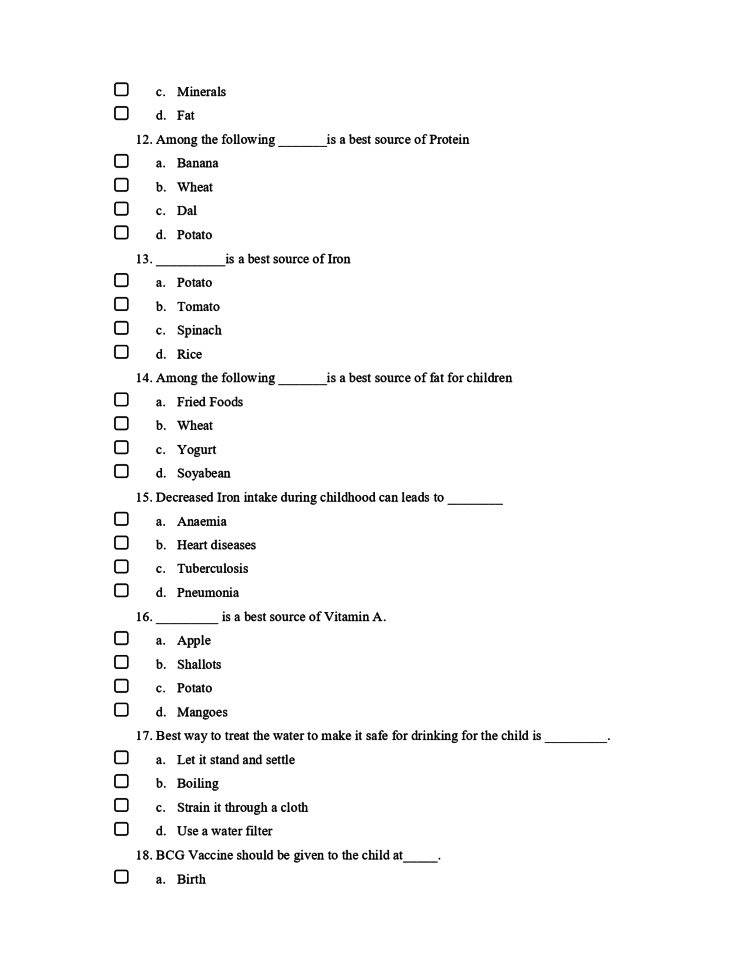
Page 5

**Figure 9:**
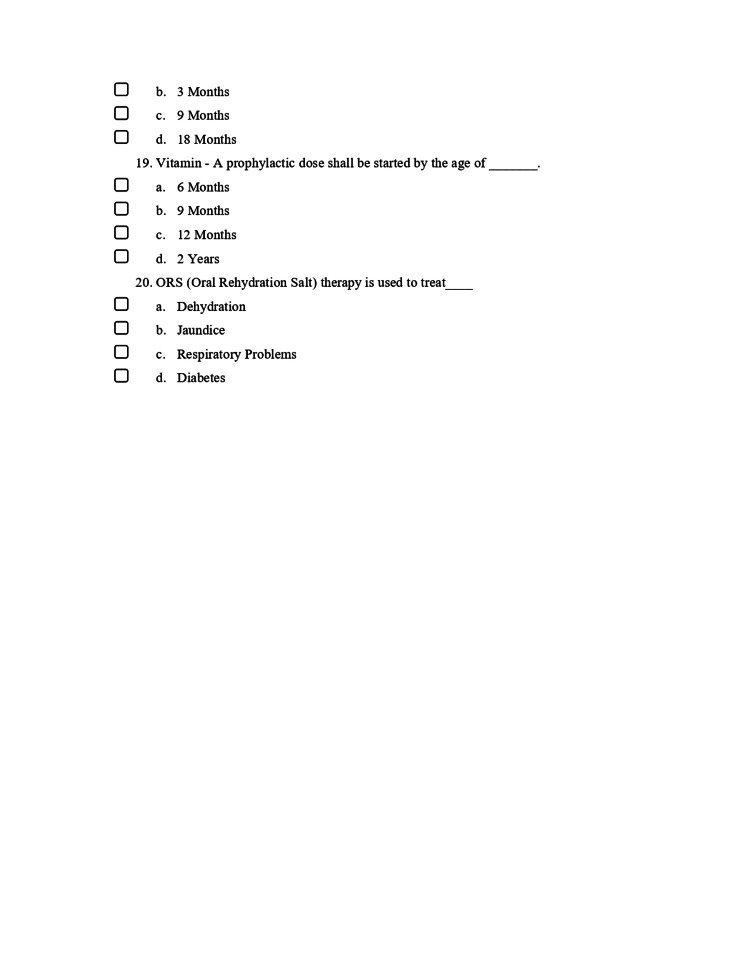
Page 6

**Figure 10:**
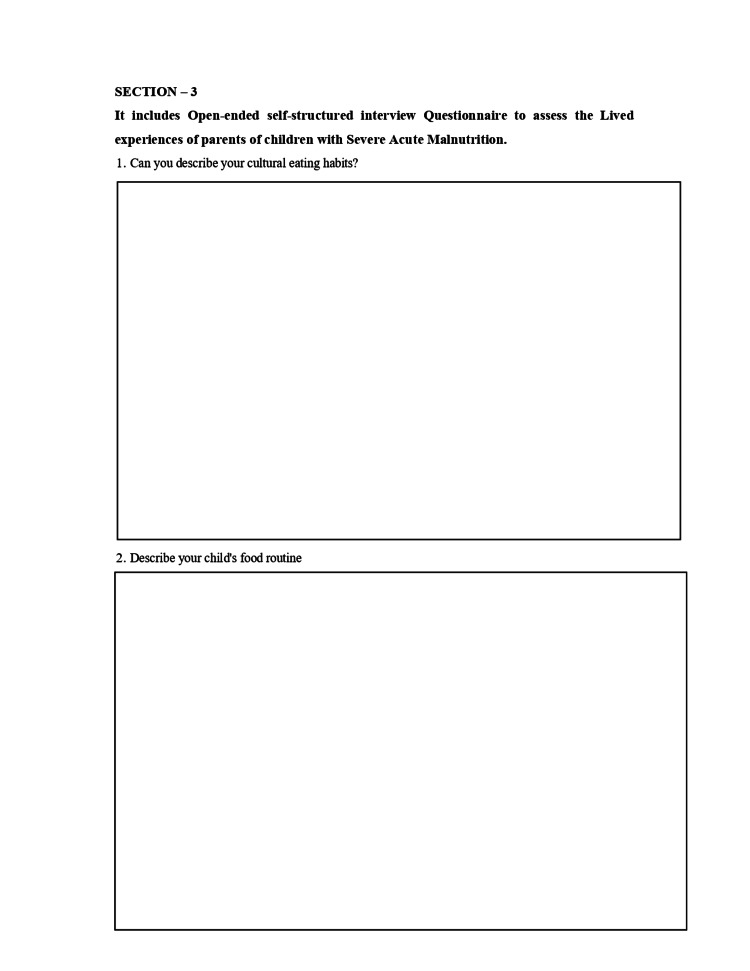
Page 7

**Figure 11:**
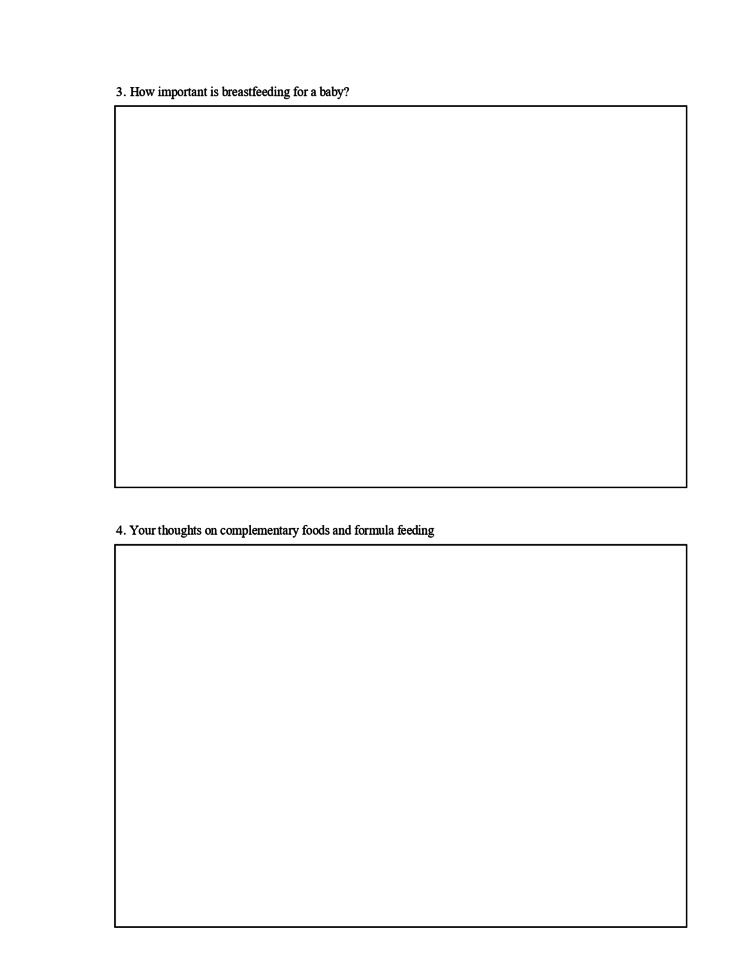
Page 8

**Figure 12:**
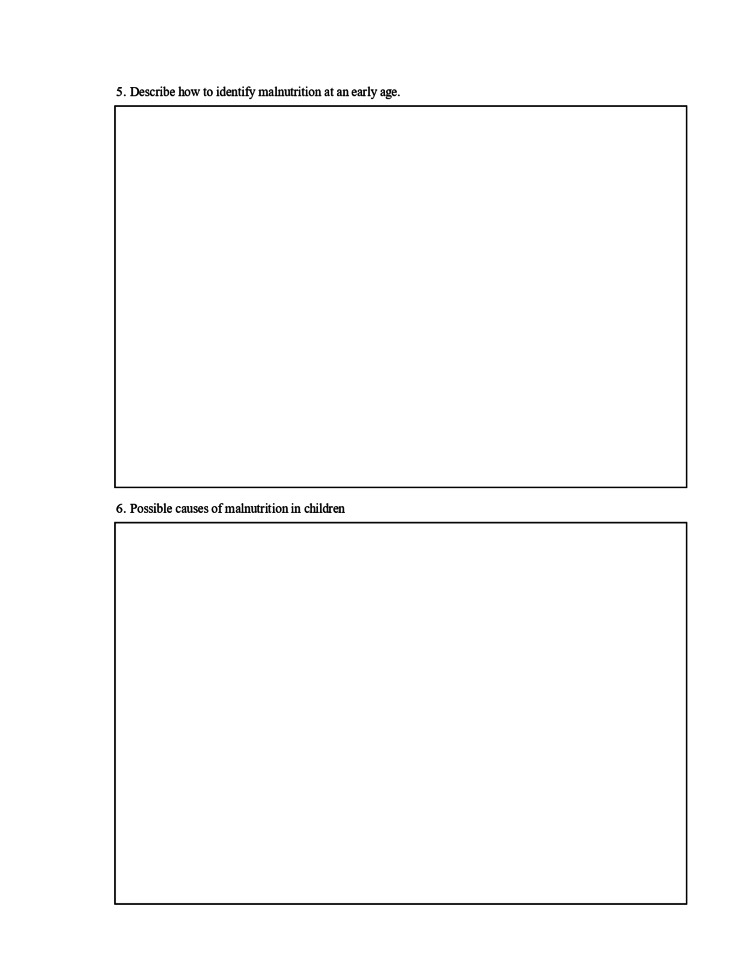
Page 9
